# Normal variation in thermal radiated temperature in cattle: implications for foot-and-mouth disease detection

**DOI:** 10.1186/1746-6148-7-73

**Published:** 2011-11-21

**Authors:** John Gloster, Katja Ebert, Simon Gubbins, John Bashiruddin, David J Paton

**Affiliations:** 1 Atmospheric Dispersion Group, Met Office, Fitzroy Road, Exeter EX1 3PB, UK; 2 Institute for Animal Health, Pirbright Laboratory, Ash Road, Pirbright, Woking, Surrey GU24 0NF, UK

## Abstract

**Background:**

Thermal imagers have been used in a number of disciplines to record animal surface temperatures and as a result detect temperature distributions and abnormalities requiring a particular course of action. Some work, with animals infected with foot-and-mouth disease virus, has suggested that the technique might be used to identify animals in the early stages of disease. In this study, images of 19 healthy cattle have been taken over an extended period to determine hoof and especially coronary band temperatures (a common site for the development of FMD lesions) and eye temperatures (as a surrogate for core body temperature) and to examine how these vary with time and ambient conditions.

**Results:**

The results showed that under UK conditions an animal's hoof temperature varied from 10°C to 36°C and was primarily influenced by the ambient temperature and the animal's activity immediately prior to measurement. Eye temperatures were not affected by ambient temperature and are a useful indicator of core body temperature.

**Conclusions:**

Given the variation in temperature of the hooves of normal animals under various environmental conditions the use of a single threshold hoof temperature will be at best a modest predictive indicator of early FMD, even if ambient temperature is factored into the evaluation.

## Background

Foot-and-mouth disease (FMD) is a highly infectious viral disease of cloven-hoofed animals, both domestic and wild. The disease is caused by a small RNA virus, which is 28 nm in diameter and exists as seven serotypes. The disease is characterised by fever, and blisters in the mouth, on the feet and on the teats and these rupture and are associated with slobbering and lameness. Adult animals may suffer weight loss and milk production can decline significantly. Though most animals eventually recover from FMD, the disease can lead to myocarditis and death, especially in newborn animals [1]. FMD is found regularly in parts of South America, Africa, the Middle East and other parts of Asia and periodically spreads to affect normally disease free countries. It is a significant impediment to trade in livestock and their products as countries with the disease face restrictions for exporting to disease free regions. Moreover, the disease is difficult and costly to control and eradicate. The Royal Society [2] estimated that during the 2001 epidemic in the UK, in which some six million animals were culled, the losses to agriculture and the food chain were £3.1 billion and some £2.5 billion was paid by the UK Government in compensation for slaughtered animals and clean-up costs. Losses were also experienced in tourism and business directly affected by tourism; it has been estimated these were between £2.7 and £3.2 billion [3]. Two other epidemics highlight the global impact of the disease; the first a major epidemic in Argentina in 2001 and the second in Japan during 2010; in the first two thousand five hundred and nineteen herds were infected [4] and in the second two hundred and fifty (Office international des épizooties-World Organisation for Animal Health, 2010. Follow-up report No 13. Information received on 15/07/2010 from Dr Toshiro Kawashima, CVO, Animal Health Division, Ministry of Agriculture, Forestry and Fisheries, Tokyo, Japan. [online] http://www.oie.int/wahis/public.php. [consulted July 2010]).

Early identification of animals infected with FMD virus is vital if disease outbreaks are to be rapidly diagnosed and controlled. Thorough screening to identify signs of FMD is time consuming and labour intensive since it requires the capture and restraint of suspect animals for clinical examination. This can be particularly difficult in some situations, for example where animals are at pasture, are difficult to handle or are present in very large numbers. Animals with FMD often develop a fever with temperatures in excess of 40°C and vesicular lesions around the coronary band, in and around the mouth and on the mammary gland. The vesicular lesions are associated with local inflammation giving rise to an increase in skin temperature which can be detected by palpation [1]. On their own, these temperature changes are not pathognomonic for FMD but can be used to select animals that warrant closer examination to detect more definitive signs and/or enable sampling for confirmatory testing.

Infrared thermography (IRT) can be used to measure the heat emitted from a surface and to display and store an image and associated data. The technique has been used by the medical profession over recent years across a range of human conditions, to identify local inflammations or pyrexia [5] and in the detection of fever associated with SARS and avian influenza [6]. IRT has also been used by those involved with animal disease [7-9]. Workers at the Pirbright Laboratory of the Institute for Animal Health (IAH-Pirbright) and at the Plum Island Animal Disease Center (PIADC), USA have reported that IRT can be used to measure the temperatures of animals that need to be checked for possible onset of FMD [10-12]. These workers studied groups of animals with experimentally-induced FMD and measured temperatures (primarily around the coronary band) as disease progressed. It was found that increases in temperature associated with FMD could be detected, sometimes prior to the development of visible lesions. Unpublished work by the current authors involving five cattle, five sheep and five pigs infected with the Asia 1 strain of FMDV discovered that it was easy to measure the feet temperatures of the animals and established that there was potential for using the technique in the field. Cattle feet temperatures ranged from 18.7°C to 31.7°C, with the highest value being recorded the day before foot lesions were visible, but at the same time as the first lesion on the tongue. Prior to the first appearance of lesions temperatures were below 27°C.

To optimise interpretation of temperature measurements and to demonstrate the reliability of the technique to differentiate between infected and healthy livestock requires further IRT data from uninfected animals, kept at different ambient temperatures and under different husbandry conditions. This shortcoming is addressed here by IRT measurements and analysis from healthy cattle.

## Methods

### Experimental Design

The experimental period was divided into two phases. The first phase was designed to make observations under different IRT/animal configurations and environmental conditions, the second to examine the changes in an animal's hoof temperature over a daily cycle of activity.

In the first phase, five separate sets of temperature data were taken over a period of five months using a TIR1 imager manufactured by Fluke (temperature range -20°C to 100°C, accuracy +/- 2°C, operated at a distance of 1 to 2 m, emissivity 0.95). Two groups of nine and ten cattle initially aged 12 and 3 months old respectively, were housed in small groups in pens in an open barn at the Institute for Animal Health farm at Compton, Newbury; one half of each pen had a concrete floor and the other a slightly raised straw filled area.

**Figure 1:**
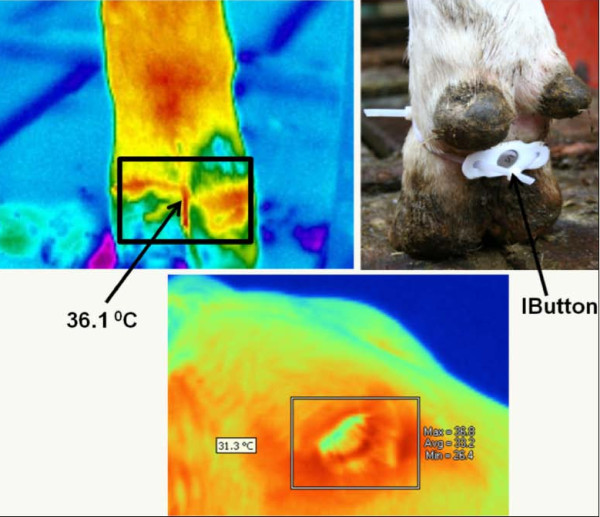
**Examples of thermal images taken during the experiment together with a photograph of an IButton attached to an animal's foot**.

Each animal in turn was restrained either by hand or in an animal crush and four IRT measurements were taken of each of the animal's feet from different aspects (front, back, lateral and medial) and a measurement was also taken of the left eye (see Figure 1 for an example). These two sites were selected because, as mentioned above, researchers working on FMD had detected an increase in temperature around the coronary band and it is hypothesised that temperatures around the eye provide a non-invasive indicator of an animal's core temperature. Other sites commonly affected by FMD lesions such as the mouth and udder are less accessible and/or only applicable to lactating animals. To investigate the link between eye temperature and body temperature each animal's rectal temperature was taken at the same time as the thermal images using a digital thermometer. The IRT images were taken twice within ten minutes from each animal to evaluate repeatability of the measurements and correct for minor variations in the angle of the imager to the animal. Ambient temperatures were measured with a Fisher Scientific model FB70357 digital thermometer.

Care was taken throughout the experiment when handling the cattle, as it was appreciated that even the simple act of gathering animals can cause an increase in stress which in turn may result in an increase in the animal's temperature.

As it was not practical to measure changes in an animal's hoof temperature over an extended period of activity using an IRT imager, a second temperature measuring device was used for this purpose (IButton data loggers, type DS1921G, temperature range -40°C to 70°C, accuracy +/- 1°C, data recording rate every second or every two seconds, manufactured by Embedded Data Systems). The IButtons were strapped to the animals' hooves as shown in Figure 1. To compare the results of IRT and IButtons, hoof temperatures were measured for two cattle. TIR1 images were taken either immediately prior to IButton attachment, simultaneously with attachment, but for another foot, or immediately after the IButton was removed. Identical readings from the two instruments were not expected as both devices measure temperature in different ways (TIR1-radiative and IButton-thermal contact). However, it was anticipated that similar trends could be detected using both sensors.

The final phase of the work was to investigate changes of hoof temperature as a function of activity. These were established using IButtons and a video security camera (Solidex Day Night DomeCam Varifocal Lens combined with a Solidex 4 channel DVR) placed above the pen holding two of the cattle (chosen for ease of visual recognition). IButtons were attached to the two hind feet of the cattle and data recorded at a frequency of once or twice per minute for a period of approximately twenty hours. Air temperatures were recorded using an IButton suspended in free air close to the animal pen. The experiment was done twice.

TIR1 images were processed using Smart View Software (V2.1.0.10), supplied by Fluke. For each image, an area of approximately 2 cm^2 ^above and below the coronary band was selected and the maximum temperature within this area (see Figure 1) was recorded and transcribed to an Excel spreadsheet for subsequent statistical analysis. Additionally, an area at least 10 cm above the hoof was selected to determine whether a ratio between hoof and more proximal leg surface temperature could help compensate for hoof temperature changes caused by ambient temperature changes. An area of approximately 2 cm^2 ^around the eye was selected for analysis and the hottest temperature within this area, including the eye itself, was recorded (see Figure 1).

For comparing IButtons and IRT, the area covered by the attached IButton was selected and the average temperature within this area recorded and further analysed in an Excel spreadsheet. IButton data was analysed with TempIT software supplied by Signatrol (Version 4.1.8) and the data transferred to the master Excel spreadsheet. To determine the animal's movements the security camera images were replayed and activity allocated into one of four categories (lying down, standing on deep straw, standing on concrete and outside of the holding pen). The date and time for each change in activity category was recorded for comparison with the IButton data.

### Statistical analysis

Two separate analyses of the data were carried out to assess: (i) the repeatability of thermal image measurements taken sequentially within a ten minute interval; and (ii) the potential for defining a threshold temperature above which cattle would be considered abnormal based on IRT.

The repeatability of thermography was assessed by computing the difference in temperature as measured by corresponding images (i.e. for the same hoof with the same aspect on the same day) for each animal and determining whether the median differed significantly (*P *< 0.05) from zero using a Wilcoxon signed rank test.

The potential for defining a threshold temperature to identify unhealthy cattle based on IRT was examined using a Bayesian hierarchical model, which incorporates between-animal variation and facilitates predictions outside the data which allow for parameter uncertainty. In this approach, the observed hoof temperature (*T_jk_*) for the *j*th observation on animal *k *was described by,

$$\begin{array}{c} T_{jk}\sim N\left({\text{μ}}_{jk}^{\left(T\right)},{\text{σ}}_{e}^{2}\right)\\ {\text{μ}}_{jk}^{\left(T\right)}=b_{0}^{\left(k\right)}+\underset{i}{\sum }b_{i}^{\left(k\right)}X_{ijk}, \end{array}$$

where ${\text{μ}}_{jk}^{\left(T\right)}$ is the expected hoof temperature, ${\text{σ}}_{e}^{2}$is the error variance, the $b_{i}^{\left(k\right)}$s are parameters and $X_{ijk}$is the value of the *i*th factor (e.g. hoof, aspect or ambient temperature) for the *j*th observation on animal *k*. Between-animal variation was modelled by assuming that the parameters for each animal are drawn from higher-order distributions, such that,

$$b_{i}^{\left(k\right)}\sim N\left({\text{μ}}_{b_{i}},{\text{σ}}_{b_{i}}^{2}\right).$$

Non-informative priors were used for the higher-order parameters: diffuse Normal distributions for the ${\text{μ}}_{b_{i}}$s and diffuse gamma distributions for the ${\text{σ}}_{b_{i}}$s. Parameters in the model were estimated using Markov chain-Monte Carlo methods implemented in WinBUGS [13]. Two chains of 50,000 iterations were run for each model, with the first 10,000 iterations discarded to allow for burn-in of the chain. Each chain was then thinned by sampling every tenth iteration to reduce autocorrelation amongst the samples.

The fits of different models were compared using the deviance information criterion (DIC) [14]. Posterior predictions for the expected hoof temperature as a function of ambient temperature were generated by sampling from the joint posterior density for the higher-order parameters. A range of percentiles of the resulting distribution were used to define thresholds for identifying abnormal animals and the specificity of a classification scheme based on these thresholds (essentially the proportion of animals below the threshold) was assessed.

## Results

In the first phase of the experiment, between July and November 2009, around two thousand three hundred thermal images of cattle hooves were taken. During these experiments, ambient temperatures ranged from 10°C to 24.8°C and general weather conditions from a warm summer's day through to cold and damp winter conditions.

**Figure 2:**
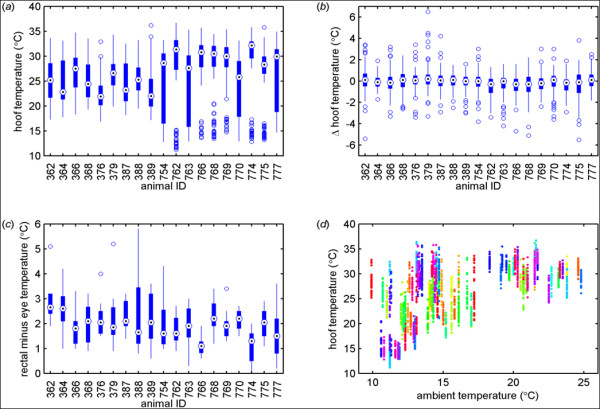
**Summary of data extracted from thermal images**. (a-c) Box-and-whisker plots showing: (a) distribution of hoof temperatures for each animal; (b) differences in hoof temperature measured by replicated thermal images for each animal; and (c) differences between rectal and eye temperature for each animal. Each plot shows the median (target), interquartile range (blue box), 1.5-times the interquartile range (whiskers) and any outliers (circles). (d) Scatter plot showing hoof temperatures and their dependence on ambient temperature. The plot shows the data for all animals, with each animal indicated by a different coloured point.

**Figure 3:**
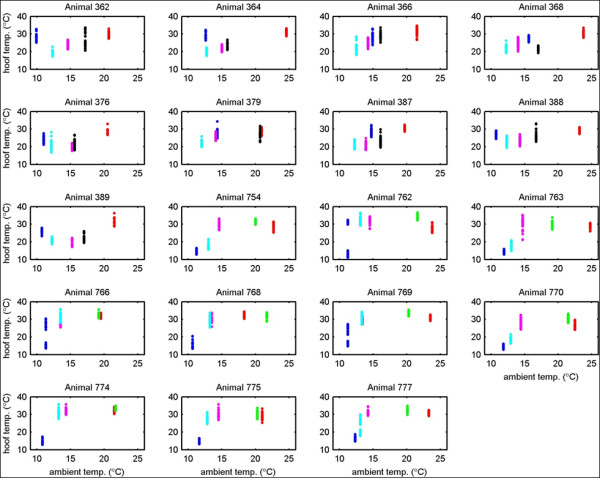
**Scatter plots showing hoof temperatures and their dependence on ambient temperature for the 19 animals in the study**. Colours in the plots indicate the date of measurement: 23/07/2009 (red); 26/08/2009 (green); 04/09/2009 (black); 08/10/2009 (blue); 22/10/2009 (magenta); and 19/11/2009 (cyan). The data for each day comprise all observations (up to 33) of all hooves from all camera aspects.

Hoof temperatures measured by IRT ranged from approximately 10°C to 36°C (Figure 2a) and depended markedly on ambient temperature (Figures 2d &3). Furthermore, the variability in hoof temperatures was greatest at lower ambient temperatures (Figure 2d). The median range in hoof temperatures for individual animals on a given day was approximately 6°C, but in some cases it was > 12°C (Figure 3). This range often reflected one hoof or side being markedly warmer than the other (for example, animals 362, 762, 766 and 769), but sometimes there was no clear explanation for the difference (for example, animals 763 and 777).

Differences in hoof temperature between corresponding images recorded on the same day were typically small (Figure 2b) and did not differ significantly (*P *> 0.05) from zero for 13 (out of 19) animals. For six animals (animals 379, 762, 766, 768, 769 and 774), the median difference in repeated observations was significantly (*P *< 0.05) different from zero, though the median difference in each case was only a fraction of a degree (range: -0.3°C to 0.2°C). Eye temperature measured by IRT provided a reasonable proxy measure for body temperature, with eye temperatures approximately 2°C lower than rectal temperature (Figure 2c) and not significantly affected by ambient temperature (*P *> 0.05).

An adequate model to describe the hoof temperature data included ambient temperature (°C), hoof (coded as: front-left, front-right, hind-left and hind-right) and camera aspect (coded as: front, lateral, medial and rear) (Table 1); removing any of these terms from the model significantly worsened model fit (full model: DIC = 17270.6; removing ambient temperature: DIC = 18846.8; removing hoof: DIC = 17309.8; removing aspect: DIC = 17297.2). Adding extra terms to the model improved the model fit, often markedly so; for example, a quadratic term for ambient temperature (DIC = 14730.3) or eye temperature as a normalising factor (DIC = 16648.1). However, this was at the expense of the resulting model being poor as a predictive tool, because the variance for the higher-order parameters needed to be so large to incorporate the observed differences amongst animals. Accordingly, the adequate model was used in subsequent analyses.

**Table 1:** Posterior estimates for the higher-order parameters in the model of hoof temperature.

Factor	Parameter	Mean	Median	95% credible limits	
				
				Lower	Upper
intercept	mean	14.03	14.04	11.60	16.37
	s.d.^†^	4.88	4.76	3.40	7.09
ambient temperature	mean	0.78	0.78	0.64	0.93
	s.d.	0.29	0.29	0.20	0.43

hoof (front-left is baseline)					
front-right	mean	0.24	0.24	-0.51	1.00
	s.d.	1.35	1.32	0.83	2.09
hind-left	mean	-0.06	-0.06	-0.48	0.37
	s.d.	0.26	0.19	0.03	0.83
hind-right	mean	-0.04	-0.04	-0.46	0.38
	s.d.	0.21	0.14	0.03	0.70

camera aspect (front is baseline)					
lateral	mean	-0.94	-0.94	-1.34	-0.53
	s.d.	0.13	0.10	0.03	0.42
medial	mean	-1.19	-1.20	-1.60	-0.77
	s.d.	0.12	0.09	0.02	0.36
rear	mean	-0.69	-0.70	-1.10	-0.28
	s.d.	0.13	0.10	0.03	0.43

^† ^s.d.: standard deviation (reflecting between-animal variability in the parameter)

The analysis indicated that there was variation in temperature amongst hooves on the same animal, but these differences were not systematic between animals, as evidenced by means for the hoof parameters which do not differ significantly from zero, but which have a high standard deviation (Table 1). Camera aspect did influence hoof temperature measurement, with images taken from the lateral, medial or rear aspect being around 1°C lower than those taken from a front aspect (Table 1). However, ambient temperature had the greatest impact on hoof temperature (Figures 2d &3; Table 1).

**Figure 4:**
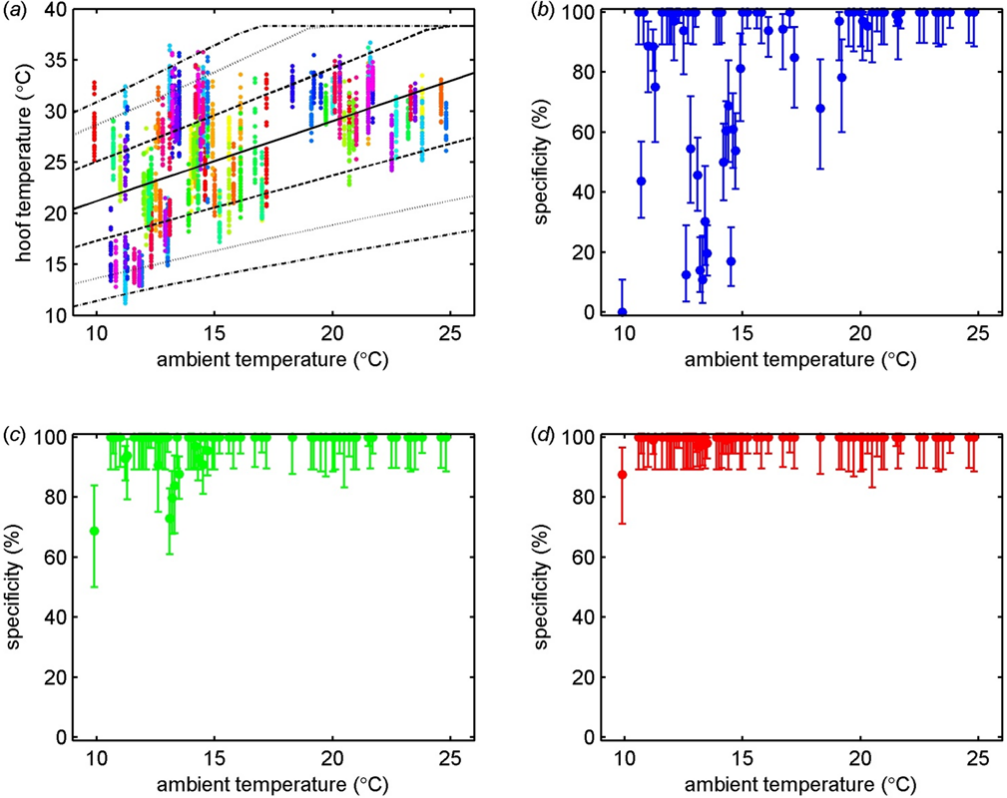
**Posterior predictions for hoof temperature and the definition of a threshold temperature for healthy cattle**. (a) posterior median (solid line), 25th and 75th percentiles (dashed lines), 10th and 90th percentiles (dotted line) and 5th and 95th percentiles (dash-dotted lines) for hoof temperature at different ambient temperatures based on a model of hoof temperature including ambient temperature, hoof and aspect. The points show the observed hoof temperatures, with each animal indicated by a different coloured point. (b-d) Specificity when classifying cattle as healthy based on a threshold defined by the (b) 75th, (c) 90th or (d) 95th percentile for the posterior predictions of hoof temperature. Each plot shows the estimated specificity (circles) and 95% confidence limits (error bars).

By sampling from the joint posterior density for the higher-order model parameters (and integrating out the effects of hoof and camera aspect) it was possible to generate predictions for hoof temperature as a function of ambient temperature. The 75th, 90th and 95th percentiles for these predictions were then used to define thresholds by which to identify healthy cattle, with a further refinement that the maximum threshold temperature was set equal to the mean rectal temperature for the animals (38.3°C) (Figure 4a; Table 2). The specificity of a classification scheme based on these thresholds was investigated. For a threshold based on the 75th percentile, the predicted specificity was low, especially at ambient temperatures below 20°C (< 80% specificity, Figure 4b). The specificity was improved by setting a threshold based on the 90th or 95th percentile with specificity > 90% predicted above temperatures of 15°C and 10°C respectively (Figure 4c, d).

**Table 2:** Threshold hoof temperatures which could be used to identify infected cattle at different ambient temperatures.

Ambient temperature (°C)	Threshold hoof temperature (°C)		
	
	75th percentile	90th percentile	95th percentile
9.0	24.2	27.7	29.8
10.0	25.1	28.6	30.9
11.0	26.0	29.6	32.0
12.0	26.9	30.6	33.1
13.0	27.8	31.7	34.2
14.0	28.7	32.7	35.3
15.0	29.6	33.8	36.4
16.0	30.5	34.9	37.6
17.0	31.4	36.0	**38.3**
18.0	32.4	37.0	**38.3**
19.0	33.3	38.1	**38.3**
20.0	34.2	**38.3**	**38.3**
21.0	35.1	**38.3**	**38.3**
22.0	36.1	**38.3**	**38.3**
23.0	37.0	**38.3**	**38.3**
24.0	38.0	**38.3**	**38.3**
25.0	**38.3**	**38.3**	**38.3**
26.0	**38.3**	**38.3**	**38.3**

**Bold**: mean animal rectal temperature measured during the study

A simple comparison between the TIR1 and three IButtons, on a shaded uniform temperature carpet tiled floor revealed that both instruments recorded similar temperatures with the TIR1 being warmer than the IButton by 0.1 to 1.4°C (IButton no./TIR1/IButton: 1/24.3/23.0; 2/24.3/22.9; 2/23.6/22.9; 3/24.3/23.5; 23.6/23.5°C). It was also established that the IButtons, given a sudden temperature change of 15°C took fifteen minutes to reach equilibrium.

Table 3 presents the results from the comparison between the IButton and TIR1 for two animals on two separate days. The data show that temperature measured by the IButton approximately fifteen minutes after attachment and just before removal relates well to the average temperatures measured by the TIR1. The IButton temperatures were consistently warmer than the average temperature measured by TIR1 (average temperature differences 5.3, 5.8, 4.4 and 1.9°C). This trend was observed for all data collected during the comparison of the IButton and TIR1. The IButton as well as the TIR1 record sudden temperature changes equally well as seen on one occasion where the average temperatures for all feet for one animal measured by the TIR1 were 10.2/11.2/12.3 and 9.6°C before IButton attachment; whereas after the removal of the IButton average temperatures for all legs measured by the TIR1 were only very slightly raised (0.1-1.3°C) apart for one foot where the temperature was raised by 14.7°C. The IButton recorded 14°C and 15°C for three out of the four legs at fifteen minutes after attachment and before removal, but recorded temperatures of 18°C after fifteen minutes and 29.5°C before removal for the leg with the raised temperature (data not shown).

**Table 3:** Hoof temperatures measured using TIR1 and IButton

Date	Cattle ID	Sample no.*	TIR1 (°C)				IButton** (°C)		Ambient temp (°C)
				
			FL	FR	RL	RR	RL	RR	
8/3/2010	770	1	11.1	10.4	9.5	10.1			5
			11	10.3	11.1	8.3			
			9.6	8.3					
		2	13.1	13.5			15.9 to	16.5 to	
			12	11.6			17.5	17	
		3			12.7	15.8			
					13.3	13.1			

8/3/2010	777	1	9.9	9.1	8.5	7.7			3.5
			10.2	9.2	8.7	5.9			
		2	11	8.8			15.5 to 15.0	15.0 to 15.5	
		3			12.5	10.3			
					8.5	12.6			

22/3/10	770	1	26.9	26.2	27.1	25.1			11
			23.8	25.7	27.1	22.4			
			25.3						
		2	18.3	18.4			26.5 to 29.0	25.5 to 30.0	
				16.4					
		3			24.2	20			
					24.4				

22/3/10	777	1	26.6	26.1	26	26.3			11
			24.7	24.4	26.6	26.9			
		2	20.2	18.6			28.5 to 25.0	28.5 to 23.5	
		3			25.3	23.9			
					24.1	22.8			

* Observation 1 taken prior to IButton being placed on the animal's rear leg, Observation 2 with IButton attached and Observation 3 immediately after the IButton was removed.

** IButton temperatures recorded fifteen minutes after being attached to the animal's foot (to allow for the sensor to reach equilibrium)

**Figure 5:**
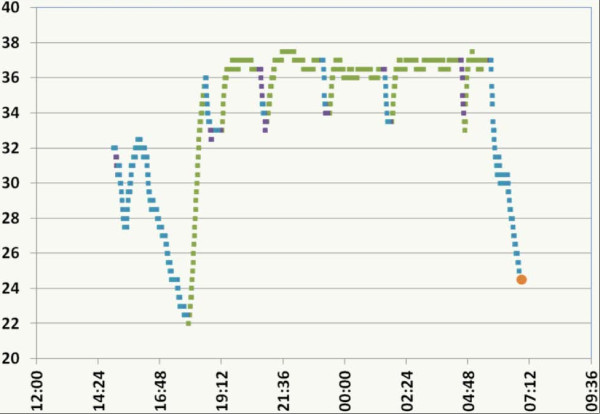
**IButton temperature data (°C) overnight 22/23 March 2010**. Green = cow lying down, blue = standing on concrete, purple = standing on deep straw, orange = animal taken out from enclosure.

The extended measurement period using both the IButton and security surveillance camera showed that the animals' hoof temperatures varied by as much as 20°C depending upon a combination of activity and ambient air temperature. A typical analysis is given at Figure 5 where it can be seen that when the animal was standing on the concrete temperatures were much lower than when it was lying down in the straw with its feet tucked under its body. This effect was consistent in each of the animals whose temperatures were measured. Maximum temperatures of 38°C were recorded and this was very close to the animal's rectal temperature.

## Discussion

To detect inflammatory conditions such as FMD affecting cattle feet, IRT needs to be able to identify abnormal surface temperature elevations. This raises the challenge of being able to distinguish such elevations from the spectrum of variability found in uninfected animals. Similar challenges affect the use of the technique in screening human subjects, for instance for pyrexia at airports [6].

Two approaches can be envisaged for FMD. First, IRT could prove very useful if a threshold temperature were to be established above which a foot temperature triggers a suspicion of an inflammatory condition. This approach has been suggested by Rainwater-Lovett [12]. However, the current study shows that this technique may be too simplistic in its approach as an animal's hoof temperature is significantly affected by ambient temperature and posture/activity. Thermal image data reported by Bashiruddin [11] from FMDV-infected cattle were compared with the thresholds shown in Table 2 to determine if these thresholds could provide a basis for early stages of FMD infection to be detected. At the ambient isolation facility temperature of ~16°C, Table 2 suggests that hoof temperatures of 30.5°C (75^th ^percentile), 34.9°C (90^th ^percentile) and 37.6°C (95^th ^percentile) would indicate an elevated temperature indicative of infection. However, of the five animals which became infected, only one showed a temperature above 30°C (two hooves) and this was when vesicular lesions were visible. Although definitive conclusions will require study of greater numbers of infected animals, these results suggest that the threshold temperatures determined in the present study will result in a low sensitivity, unless specificity is reduced.

An alternative approach is for the operator to use IRT to identify hot-spots. These are identified as either part or all of a hoof that is hotter than the surrounding skin or hotter than other feet. In this approach, it is relative rather than absolute temperatures that matter. Previous studies [11,12] have demonstrated that areas of raised temperature on an animal's hoof can be detected. To investigate this approach, a "blind test" was conducted using forty four thermal images from six cattle either before infection or in the early stages of FMD [11]. One of the authors was invited to categorise the images as "not a concern", "unlikely to be infected", "possibly infected", "suspicious" or "highly suspicious". Once an animal displayed clinical signs evident upon close physical examination, it was considered infected and it was excluded from further analysis the day afterwards, since temperatures of the feet often decline within a day or two of the formation of vesicles even if ruptured lesions remain evident. The results from this pilot revealed a 70% sensitivity (7 out of 10 images) and 79% specificity (scoring possibly infected and above as positive) (27out of 34 images) or 30% sensitivity (3 out of 10 images) and 94% specificity (scoring suspicious and above as positive) (32 out of 34 images). Whilst these results are encouraging, further work using images collected from a larger number of infected animals is needed before a conclusion can be reached concerning the merits of this approach.

This study has been completed under ideal field conditions. The situation in the field is likely to be less favourable. For example the animal's feet may be wet, covered in grass, muddy or covered in faeces. These variables need to be studied in more detail before IRT can be used with confidence to detect FMD in the field. Other parts of the body affected by inflammation in FMD, such as the mouth are not readily visualised by an infrared camera, whilst changes in the udder are limited in application to female dairy breeds. The use of IRT eye measurements seems a promising method to measure body temperature and therefore merits further evaluation in animals affected with FMD and other pyrexic conditions.

If IRT technology is to be useful in the field it has to be both technically capable of distinguishing between infected and non infected animals and be a cost effective diagnostic tool. In the field two scenarios are likely; the first where animals are housed or can be easily corralled and are readily accessible at close range and the second where they are at pasture and less easy to gather. In the first instance the current cost of an IRT camera will be in the range £2-10 k but in the second, where the equipment is required to operate at longer ranges, it is likely that a more powerful telephoto lens would be required. The cost of this significantly increases the price of the equipment possibly up to £20 k.

## Conclusions

The study has identified that an animal's hoof temperature is influenced by its activity prior to the point at which thermal screening is performed. Consequently, a period of acclimatisation is required prior to an image being taken. This is particularly the case if the animal has been lying down with its feet tucked under its body.

The work has shown that IRT images of an animal's eye temperature may be a useful proxy for core temperature and could be used to detect pyrexia as an indicator for selecting animals for closer examination. This conclusion supports the observation by Dunbar [10] who compared high quality thermograms of the eye (n = 16) to body temperature and found them not to be different (p = 0.19). However, further work is required with animals infected with FMDV to confirm this.

## Competing interests

The authors declare that they have no competing interests.

## Authors' contributions

JG and DJP were responsible for study design. JG, KE, DJP and JB were responsible for the field work, KE for thermography data analysis and SG for statistical analysis and modelling. DJP was responsible for conducting the "blind test". All authors read and approved the final manuscript.
